# Effect of muscle stretching and isometric exercises on quality of life in children undergoing regular hemodialysis

**DOI:** 10.1007/s00467-024-06398-2

**Published:** 2024-06-27

**Authors:** Shimaa Hassan Khalf-allah, Hekmat Ebrahim, Ahlam Badawy, Hend Sayed

**Affiliations:** 1 Pediatric Nursing Department, Faculty of Nursing, Assiut University, Asyut, Egypt; 2 Pediatrics Department, Faculty of Medicine, Assiut University, Asyut, Egypt

**Keywords:** Children, Hemodialysis, Isometric exercises, Muscle stretching, Quality of life

## Abstract

**Background:**

Chronic kidney disease (CKD) is a prevalent health issue that can have detrimental effects on the quality of life (QoL) of children. Nevertheless, with adequate management and support, many children with CKD can have satisfying lives. The study aimed to investigate the effect of muscle stretching and isometric exercises on QoL of children undergoing hemodialysis.

**Methods:**

Sixty-eight children aged 6–18 years with kidney failure undergoing hemodialysis at Assiut University Children Hospital were included. They were randomly assigned to two groups. The study group received a 40-min exercise program three times per week for 2 months, while the control group received routine hospital care. For outcome measures, two tools were used: a simple questionnaire sheet for personal and medical data and PedsQL™ scale.

**Results:**

After 2 months of exercise, it was shown that most children in the study group (66.7%) had good QoL, in contrast to only 3.3% in the control group, with a highly statistically significant variation between the two examined groups pertaining to the health-related QoL scale (*P* value = 0.001) after exercise.

**Conclusion:**

The intensity of care for children on hemodialysis has a distinguished impact upon their quality of life. The implementation of muscle stretching and isometric exercises during hemodialysis represents an important aspect of such care that may be associated with significant improvement in all domains of QoL. Children undergoing hemodialysis need well-organized programs that cover all physical and psychological aspects with smart time manipulation and increased attention from their staff.

**Graphical Abstract:**

**Figure:**
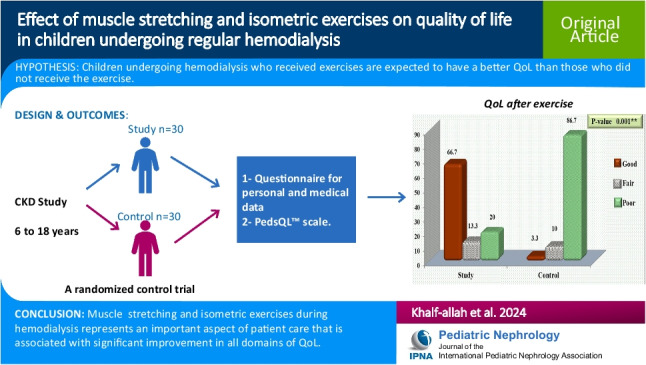
A higher resolution version of the Graphical abstract is available as Supplementary information

**Supplementary Information:**

The online version contains supplementary material available at 10.1007/s00467-024-06398-2.

## Introduction

Chronic kidney disease (CKD) is a significant global health problem, with a high mortality rate, particularly among children [1]. Kidney replacement therapy is necessary to treat this disease, with hemodialysis as one of the main replacement therapies [2].

The majority of research evidence points out that children undergoing hemodialysis have relatively poor physical function, impaired performance, and weak muscle strength. These factors are associated with poor quality of life (QoL) and amplified mortality rate in pediatrics [3]. Studies have also shown that children on hemodialysis may also experience emotional and social difficulties, such as isolation and anxiety, and may miss school or fall behind in their studies due to their condition and treatment. Therefore, it is important for healthcare providers and caregivers to take into account the impact of the disease and hemodialysis treatment on a child’s physical fitness and psychological well-being and to provide adequate support and management to improve their QoL. This may include exercise programs and educational activities to help children and their families manage their condition and maintain a good QoL [4].

Exercise and physical activity have been identified as playing a crucial part in promoting health and well-being in children undergoing hemodialysis. Exercise raises muscle tissue flexibility by promoting physiological progressions such as skeletal muscle restoration and the regulation of myostatin action [5]. In particular, muscle stretching and isometric exercises are considered two forms of physical activity that can be used safely in children undergoing hemodialysis and offer numerous benefits [6]. Stretching and isometric exercises can relax the muscles, improve concentration, reduce stress tension, reduce muscle pain and soreness, increase elastic muscle tone, and lower lactic acid build-up in the muscles. This can improve physical function, child health, and finally QoL [7].

Even though the benefits of physical exercise are known, children undergoing hemodialysis often avoid engaging in a variety of physical activities because of being afraid of complications. The lack of physical exercise and sedentary lifestyle in children contribute to behavioral and social problems as well as reduced QoL. Therefore, the chief aim of this research was to assess the effect of muscle stretching and isometric exercises on the QoL of children on hemodialysis and to teach them how to regularly practice muscle stretching and isometric exercises in order to enhance their QoL.

### Hypothesis

Children undergoing regular hemodialysis who received muscle stretching and isometric exercises are expected to have a better QoL than those who did not receive the exercise.

### Operational definitions

Quality of life is the overall well-being and satisfaction that individuals experience in their daily lives. It encompasses various aspects, including physical health and emotional well-being, school performance, and social relationships. The operational definition of quality of life often involves the use of validated questionnaires, surveys, or scales that capture these dimensions and provide quantifiable data for analysis and comparison.

Physical exercise is an exercise that is done for children undergoing hemodialysis and includes a variety of techniques such as stretching exercise (which involves the deliberate elongation of muscles to improve flexibility and range of motion, and prevent muscle tightness or injury) and isometric exercise (which involves muscle contractions without joint movement).

## Methods

### Study design

A randomized control trial was undertaken over a period of 2 months, commencing on the first day of July (2022) and concluding on the first day of September (2022).

### Study population

**Fig. 1:**
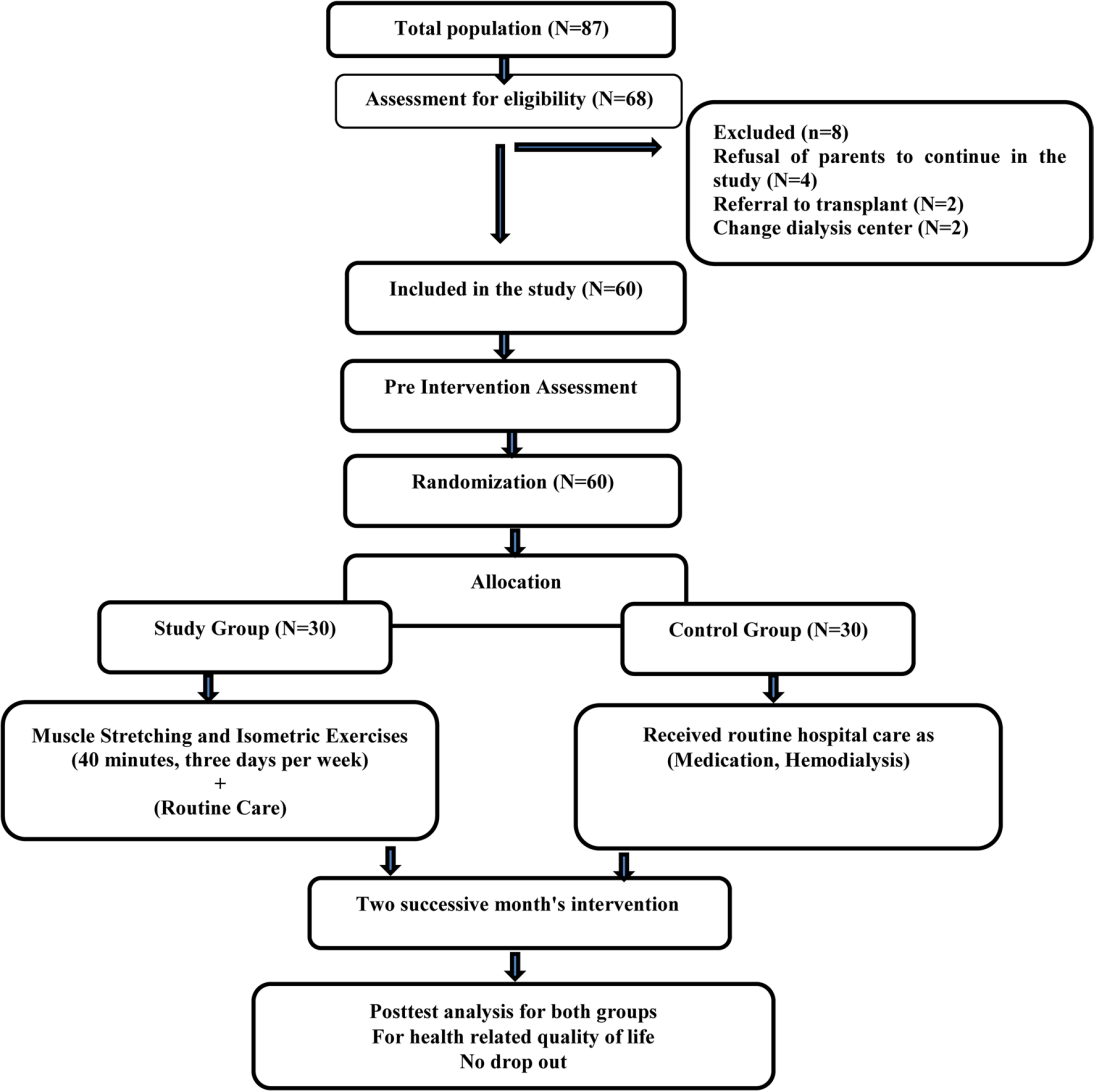
Study flow chart

The study participants were 68 children from a total population of 87 children undergoing hemodialysis at the Pediatric Nephrology Unit in Assiut University Children’s Hospital aged from 6 to 18 years old. We included only children who were stable on dialysis for 2 months, free from lower extremity grafts and pathology, did not exercise for 6 months before treatment, and without a femoral dialysis catheter or internal jugular vein catheter. Then 8 children from the 68 eligible were excluded from the study because of the refusal of parents to continue in the study (*n* = 4), referral to a transplant center (*n* = 2) and change of dialysis center (*n* = 2). Such data are demonstrated in the flow chart in Fig. 1.

### Sample size calculation

The necessary sample size for the study was calculated using G Power Software 3.1.9.7. A one-way ANOVA test with fixed effects, an effect size (*f*) = 0.65, α error of 0.05, and an actual power (1–β) = 0.89 were conducted. The results indicated that a minimum of 57 children were required to ensure adequate statistical power. To account for possible dropouts, 68 children (34 for each group) were selected for the study.

### Randomization

The randomization process was initiated after 68 children undergoing hemodialysis were assessed for eligibility and baseline measurements were taken. Each child was assigned a number between 1 and 68 in serial order, and then randomization was carried out through a web-based randomizer (https://www.randomizer.org/) to allocate 34 children to each group. The study group received muscle stretching and isometric exercises plus their routine care, whereas the control group received their routine care in the form of medication and dialysis, having a healthy snack, sleep for a short time, playing short verbal games with their caregiver, and watching TV for some time.

### Outcome measures

1. *A structured questionnaire* sheet was utilized to evaluate the personal and medical data of the participating children. The personal data included the following: age, sex, birth order, educational level, parents’ education, and occupation, while the medical data consisted of the following: the duration of illness, duration of hemodialysis treatment, number of sessions per week, age of patient at diagnosis, and associated diseases.
2. *Pediatric Quality of Life (PedsQL™)* scale version 4.0 was adopted from Uneri et al. [8] and was translated into Arabic after checking its local validity. This measure was carried out at the beginning of the trial and again after a period of 2 months. The PedsQL™4.0 generic self and proxy health-related QoL questionnaires found good psychometric properties with regard to acceptability, responsiveness, validity, and reliability. This instrument appeared to be easy to use and comprehend within the target population of children. Additionally, it was used in rigorous previous research on similar populations. The PedsQL™ questionnaire consisted of 23 items that were categorized into four domains: physical (8 items), emotions (5 items), social functioning (5 items), and school performance (5 items). Each item was scored on a scale of 0–4, with 4 indicating that the child never had a problem, 3 representing that they almost never had a problem, 2 indicating that they sometimes had a problem, 1 showing that they often had a problem, and 0 signifying that the child almost always had a problem. The scoring system for each item was 0 = 100, 1 = 75, 2 = 50, 3 = 25, and 4 = 0. The total scoring system was used to categorize the QoL of the children. If a child’s score was less than 50%, their QoL was deemed poor. If their score was between 50 and less than 75%, their QoL was considered fair. Finally, if their score was between 75 and 100%, their QoL was regarded as good. The reliability and validity of (PedsQL™) measure used in this study were assessed; it had good internal consistency (α = 0.98) [8].

The researcher who collected data from patients and their parents was blinded to patient subgroups.

### Procedures

The study was designed to be conducted in four stages (preliminary, assessment, application, and appraisal phases).

#### The preliminary phase

Official authorization was obtained from the Head of the Pediatric Hemodialysis Unit at Assiut University Children’s Hospital to collect the necessary data for the study. To ensure that the research tools were clear and applicable to the study, a pilot study was conducted on 10% (6) of the participating children. Written informed consent was obtained from the parents of the children participating in the study, with assurances that the data collected would be kept confidential and used solely for research purposes.

#### Assessment phase

The researcher went to the hospital and included children who were present in the hemodialysis unit, then made a random assignment of children to the study or control group, and then made a pretest for the studied children using outcome measure tools. The PedsQL™ testing utilized age-specific testing materials to ensure that the questions and response options were appropriate for each age group. For the younger age group or children who may have difficulty reading or comprehending the questions independently, the tests were typically completed with the help of parents or caregivers. In these cases, the parents would provide the answers based on their observations and understanding of the child’s well-being.

#### Application phase

When the child was connected to the hemodialysis machine, muscle stretching and isometric exercise were conducted 3 days per week, with an interval of 36 h between sessions. Depending on the child’s exercise tolerance, each set was performed with 10 repetitions, gradually increasing to 15 repetitions. This procedure was continued for 2 months as follows:

- In the first hour, the child did not do any activity.
- Performing the stretching and isometric exercises was done by the researcher after obtaining training from a professor of physical therapy, and when the patient became competent to perform this exercise, they could do it themselves in the presence and supervision of the researcher.
- During the study, the children in the intervention group received stretching exercises in the first 20 min of the second hour of hemodialysis. This involved flexing or stretching the calf, gastrocnemius, soleus, hamstring, and quadriceps muscles at a frequency of 10 times per session.
- In the first 20 min of the third hour, the children in the intervention group received isometric exercise. This type of strength training involved contracting the muscles (such as the chest, biceps, stomach, and hip abductor and adductor) for 3–5 s and then relaxing them at a frequency of 10 times per session.
- To prevent disconnection of the needle, exercise was not performed on the body portions linked to the dialysis machine. Instead, exercise was carried out on other body parts. Furthermore, children were instructed to stop exercising and report any adverse effects to the researchers. If a patient became unstable, the exercise was stopped immediately.
- For the control group, the children received just their routine hemodialysis care in the form of having a healthy snack, sleeping for a short time, playing short verbal games with their healthcare providers, and watching TV for some time. However, they were not engaged in any definite program.
- Eight patients failed to complete the study because of a change of dialysis center, referral to a transplant center, or the refusal by their parents to continue in the study.

#### Appraisal phase

After 2 months of muscle stretching and isometric exercise, the QoL was reevaluated and matched between the two groups by another researcher who was blinded to patient groups.

### Ethical approval

Before the beginning of the trial, the local Ethical Committee at the Faculty of Nursing at Assiut University granted approval for the research proposal (IRB: 1120230303), and the study is registered (www.trialregister.nl; NCT05980351). The privacy of the participants was maintained. Written informed consent was obtained from the parents of each child after understanding that the collected data would be utilized for research purposes.

### Statistical analysis

The acquired data was subjected to statistical analysis utilizing SPSS V.26. The presentation of the data was done through tables and charts, depicting numbers, percentages, averages, and standard deviations. The Pearson correlation was employed to investigate the relationship between variables, while the chi-square test was utilized to determine the statistical significance of the findings. Additionally, the means of the variables were compared using the *T*-test. A *P* value of 0.05 was declared statistically significant.

## Results

### Participants’ demographic data

The baseline demographic data appearing in Table 1 reveals that no statistically significant difference was found among the studied children at baseline assessment, and the majority of studied subjects were male, ranging in age from 14 to 18 years. Also, no statistically significant difference was found among the studied children as regards the descriptions of hemodialysis therapy, as demonstrated in Table 2. This indicated that the two groups were matching.

**Table 1:** Demographic data of the examined children (*n* = 60)

Children’s personal data	Study group (*n* = 30)		Control group (*n* = 30)		*P* value
	*n*	%	N	%	
Child age					
6 < 10 years	3	10.0	8	26.7	0.249
10 < 14 years	11	36.7	9	30.0	
14–18 years	16	53.3	13	43.3	
Child gender					
Male	21	70.0	18	60.0	0.417
Female	9	30.0	12	40.0	
Child birth order					
1st	6	20.0	9	30.0	0.711
2nd	11	36.7	12	40.0	
3rd	7	23.3	5	16.7	
4th and above	6	20.0	4	13.3	
Child level of education					
Illiterate	3	10.0	3	10.0	0.991
Primary education	12	40.0	13	43.4	
Preparatory education	8	26.7	7	23.3	
Secondary education	7	23.3	7	23.3	
Consanguinity of parent					
Positive consanguinity	19	63.3	20	66.7	0.787
Negative consanguinity	11	36.7	10	33.3	

Chi-square test; statistically significant at *P* value < 0.05

**Table 2:** Allocation of examined children pertaining to their hemodialysis therapy (*n* = 60)

Hemodialysis therapy	Study group (*n* = 30)		Control group (*n* = 30)		*P* value
	*n*	%	*n*	%	
Cause of kidney failure					
Unknown cause	8	26.7	10	33.3	0.734
Congenital anomalies	11	36.7	12	40.0	
Glomerulonephritis	7	23.3	3	10.0	
Nephrotic syndrome	3	10.0	4	13.4	
Lupus nephritis	1	3.3	1	3.3	
Associated diseases					
Yes (diabetes mellitus, hypertension, and heart failure)	11	36.7	14	46.7	0.432
No	19	63.3	16	53.3	
Duration of hemodialysis therapy/years					
< 1 year	6	20.0	6	20.0	0.515
1 < 5 years	16	53.3	16	53.3	
5 < 10 years	8	26.7	6	20.0	
10 years	0	0.0	2	6.7	
Number of sessions per week					
Two sessions	2	6.7	3	10	0.573
Three sessions	28	93.3	27	90	
Duration of each session/hours					
3 h	0	0.0	1	3.3	0.131
4 h	29	96.7	28	93.4	
5 h	1	3.3	1	3.3	
Complications during dialysis session					
Yes	3	10	2	6.6	0.405
No	27	90	28	93.4	
If yes					
✓Nausea and vomiting	0	0.0	0	0.0	0.268
✓Dizziness, confusion, and headaches	1	33.3	0	0.0	
✓Shortness of breath and chest pain	0	0.0	0	0.0	
✓Muscle cramps	1	33.3	0	0.0	
✓Hypotension	0	0.0	1	50	
✓Hypertension	1	33.3	1	50	

Chi-square test; statistically significant at *P* value < 0.05

### Children’s QoL mean score in pre and post intervention

Table 3 illustrates the comparative data of the PedsQL™ among the study and control groups. Data of domains of PedsQL™, which include physical, emotional, social, and school function, before and 2 months after exercise, are presented as mean ± SD. Prior to intervention, there was no significant difference among the two groups across all domains and the total PedsQL™ score. An effect size of 0.000 before intervention suggests that there was no observed difference between the groups or conditions being compared. This indicates that the intervention had not yet had an impact, and the groups were similar in terms of the outcome being measured. However, after intervention, there was a significant enhancement in the total score of the PedsQL™ in the study group relating to the control group (*P* value = 0.001**) and an effect size of 0.531, which indicates a moderate-to-large difference between the groups or conditions, in which the mean score of physical function improved from 132.50 ± 119.83 to 483.33 ± 201.32, emotional functioning from 141.67 ± 173.99 to 356.67 ± 100.63, social functioning from 195.83 ± 121.25 to 419.17 ± 96.19, and school functioning from 165.0 ± 114.39 to 389.17 ± 90.66. This suggests that the exercise had a meaningful impact on domains of PedsQL™ in comparison to the control group, which had little change, which indicated that routine care without exercise did not have a significant improvement in QoL.

**Table 3:** PedsQL™ scores before and after exercise *n* = 60

Quality of life aspects	Study group (*n* = 30)	Control group (*n* = 30)	*P* value
	Means ± SD	Means ± SD	
Physical functioning			
Before exercise	132.50 ± 119.83	92.97 ± 153.75	0.255
After exercise	483.33 ± 201.32	109.38 ± 174.22	0.001**
Emotional functioning			
Before exercise	141.67 ± 173.99	130.47 ± 131.48	0.950
After exercise	356.67 ± 100.63	141.41 ± 135.24	0.001**
Social functioning			
Before exercise	195.83 ± 121.25	232.03 ± 141.17	0.291
After exercise	419.17 ± 96.19	244.53 ± 139.52	0.001**
School functioning			
Before exercise	165.0 ± 114.39	174.22 ± 122.72	0.764
After exercise	389.17 ± 90.66	181.25 ± 123.62	0.001**
Total score			
Before exercise	635.0 ± 502.26	629.69 ± 496.21	0.967
Eta squared	0.000		
After exercise	1648.33 ± 468.62	676.56 ± 520.78	0.001**
Eta squared	0.531		

*T*-test

^**^Highly statistical significant difference (*P* value < 0.001)

**Fig. 2:**
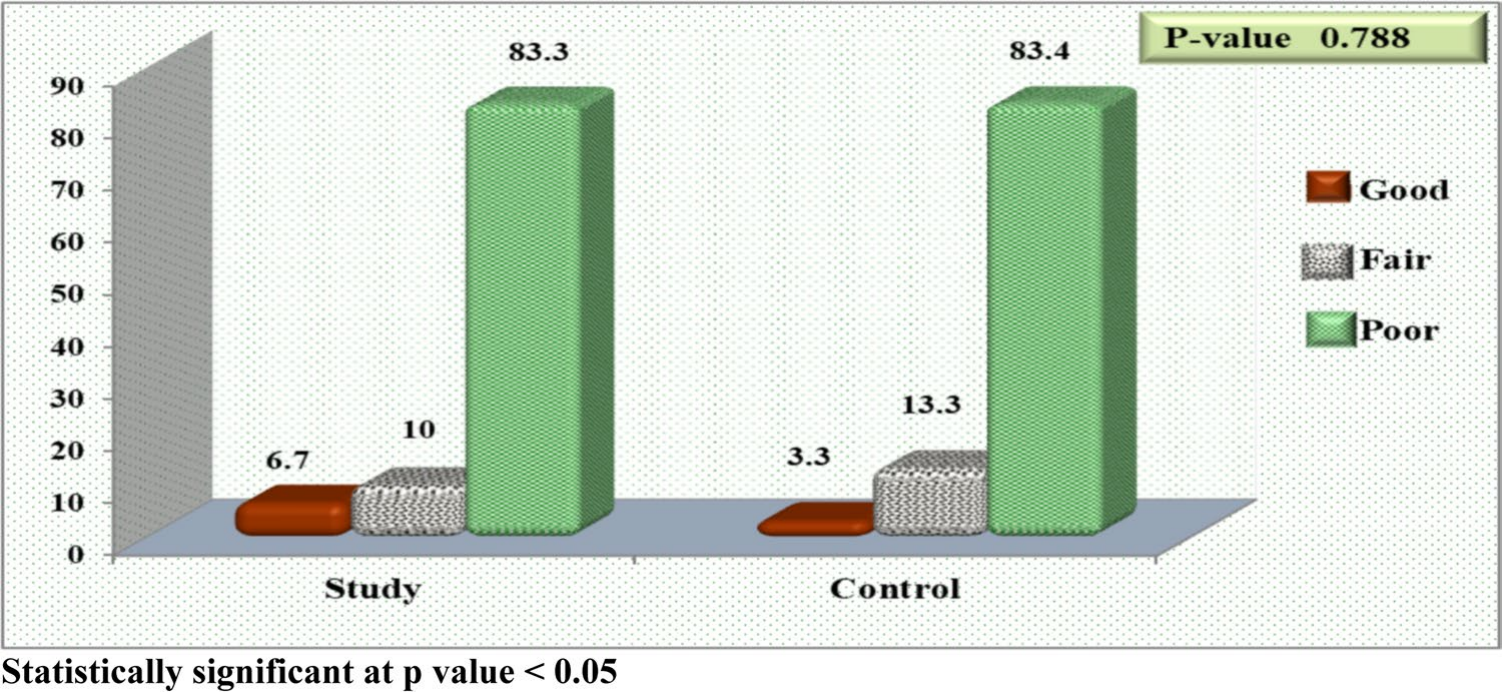
Allocation of examined children pertaining to total QoL level before exercise (*n* = 60)

**Fig. 3:**
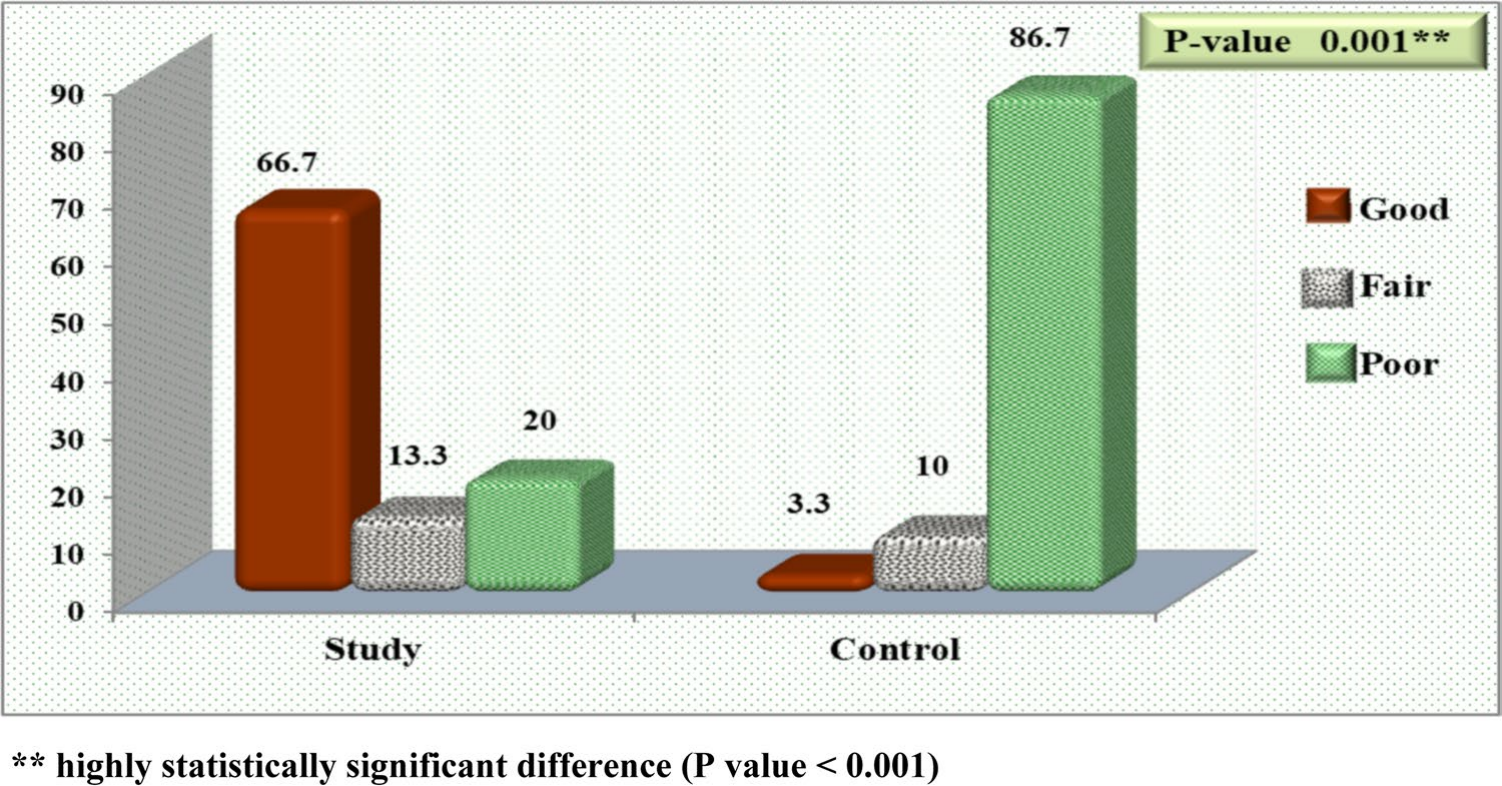
Allocation of examined children pertaining to total QoL level after exercise (*n* = 60)

Figure 2 shows there was no statistically significant difference among the examined children according to total QoL level before exercises (*P* value = 0.788), and the majority of studied children (83.3% and 83.4%) had poor QoL. However, after exercises (Fig. 3), 66.7% of the examined children in the study group had good QoL compared to only 3.3% in the control group.

**Fig. 4:**
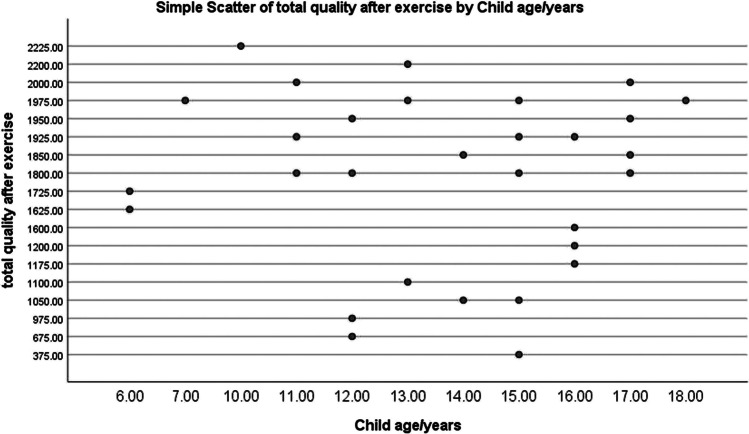
Correlation between child age and QoL in the study group after exercise (*n* = 30). Statistically significant at *P* value < 0.05

Figure 4 shows the correlation between child age and QoL in the study group after the exercise program. It was found that there was no statistically significant correlation between child age and their total QoL with *P* value = 0.47.

### Correlation between PedsQL™ score and independent variables

Finally, as presented in Table 4, there is no significant association between PedsQL™ score and personal and medical profile variables like gender, child birth order, residence, cause of kidney failure, and consanguinity of parents, but there was a significant association between the PedsQL™ score and child level of education (*P* value = 0.042) and associated diseases such as diabetes mellitus, hypertension, and heart failure (*P* value = 0.047*).

**Table 4:** Relationship between children’s demographic and clinical data and QoL in the study group after exercise program (*n* = 30)

Children’s personal data	Total QoL after exercise			*P* value
	Study group			
	Good	Fair	Poor	
	*n* (%)	*n* (%)	*n* (%)	
Child gender				
Male	15)75.0(	3)75.0(	3)50.0(	0.490
Female	5)25.0(	1)25.0(	3)50.0(	
Child birth order				
1st	3 (15.0)	2 (50.0)	1 (16.7)	0.503
2nd	6 (30.0)	2 (50.0)	3 (50.0)	
3rd	6 (30.0)	0 (0.0)	1 (16.7)	
4th and more	5 (25.0)	0 (0.0)	1 (16.7)	
Residence				
Urban	4 (20.0)	1 (25.0)	1 (16.7)	0.949
Rural	16 (80.0)	3 (75.0)	5 (83.3)	
Child level of education				
Illiterate	4 (20.0)	0 (0.0)	0 (0.0)	0.042*
Primary education	6 (30.0)	1 (25.0)	5 (83.3)	
Preparatory education	6 (30.0)	0 (0.0)	1 (16.7)	
Secondary education	4 (20.0)	3 (75.0)	0 (0.0)	
Consanguinity of parent				
Positive consanguinity	12 (60.0)	2 (50.0)	5 (83.3)	0.488
Negative consanguinity	8 (40.0)	2 (50.0)	1 (16.7)	
Cause of kidney failure				
Unknown cause	5 (25.0)	0 (0.0)	3 (50.0)	0.473
Congenital anomalies	6 (30.0)	3 (75.0)	2 (33.3)	
Glomerulonephritis	6 (30.0)	0 (0.0)	1 (16.7)	
Nephrotic syndrome	2 (10.0)	1 (25.0)	0 (0.0)	
Lupus nephritis	1 (5.0)	0 (0.0)	0 (0.0)	
Associated diseases				
Yes (diabetes mellitus, hypertension, and heart failure)	8 (40.0)	3 (75.0)	0 (0.0)	0.047*
No	12 (60.0)	1 (25.0)	6 (100.0)	

Chi-square test

^*^Statistically significant at *P* value < 0.05

## Discussion

Patients with kidney failure undergoing hemodialysis have low cardiorespiratory fitness, poor PedsQL*™*, and functional impairment compared with their age-matched controls. Exercise during hemodialysis showed improvements in cardiorespiratory fitness, physical performance, and self-reported physical function in PedsQL™ questionnaires. However, such studies have usually been performed on adult patients. Similar studies in children are not abundant, and direct translation of data from adults to children and adolescents is not applicable [9]. In this field, Wilkinson et al. concluded in their review that children with CKD are physically inactive compared to their peers. Such inactivity occurs early in the course of the disease and progressively gets worse as the disease burden increases. They also stated that there is limited evidence on interventions to increase physical activity behavior in this population, and those that have been attempted have had negligible effects [10].

The findings of the current study show that there was no statistically significant difference between the study and control groups in all domains of QoL before exercises, and the physical function domain had the lowest score, but post muscle stretching and isometric exercises, there was significant improvement in all QoL subscales and total scores in the study group, in which more than two thirds (66.7%) of examined children in the study group had good QoL in contrast to only 3.3% in the control group.

These results were consistent with Abd-Elmonem et al. [11], who conducted a study on the effects of progressive resistance exercises on QoL and functional capacity in 32 children with CKD. They reported significant improvements in QoL in the study group. However, they included a small number of CKD patients who did not start maintenance hemodialysis, and they did not evaluate the additional effect of isometric exercise in their study. Also, Soliman and Atia [12] found that there was a significant improvement in physical activity and daily life activity after 2 months of intradialytic exercise in 40 children on regular hemodialysis; however, they did not use a well-structured tool for a proper assessment of physical improvement. In addition, they did not involve psychosocial aspects in their assessment.

Feldkötter et al. [9] found that intradialytic bicycle training does not improve dialysis efficacy or physical fitness in pediatric patients. However, they considered their study underpowered because of high dropout rates.

The outcomes of our investigation align with the preceding research performed by Song and Sohng [13], which examined the impact of a 12-week gradual resistance training program on physical composition, fitness levels, and QoL. The study revealed significant enhancements in physical fitness and QoL among the participants. Furthermore, the authors proposed that gradual resistance training could be effectively included as a recurrent care strategy for patients with CKD. Also, Amri et al. [14] found that the intradialytic ergometry and stretching exercises implemented during the dialysis session were effective in reducing the severity of fatigue and cramps. However, both of these studies included adult patients with CKD, not pediatric patients.

Schaar et al. [15] evaluated the current literature on training during dialysis and found that only a few studies provide information about endurance training in pediatric hemodialysis patients. All were designed as pilot studies with small numbers, a high dropout rate, and a focus on the feasibility, acceptability, safety, and efficacy of an intra-dialytic training program. Such studies could not provide enough scientific evidence for a detailed training program. Also, exact training definitions or standard recommendations were not available. In addition, none of these studies assessed the effect of exercise on different domains of QoL for such children.

The improvements observed in the QoL domains among the study group may be attributed to the therapeutic impact of muscle stretching and isometric exercises. Stretching exercises involve the deliberate elongation of muscles to improve flexibility and range of motion and prevent muscle tightness or injury. While stretching is commonly included as a component of physical activity routines, it is not considered a vigorous form of exercise that significantly increases heart rate or causes substantial calorie expenditure. However, stretching exercises play a vital role in enhancing overall physical performance, reducing muscle soreness, and maintaining muscle balance. Isometric exercises, on the other hand, involve muscle contractions without joint movement. These exercises typically involve static positions where the muscle is under tension but does not change in length. Isometric exercises can help improve muscle strength and endurance. While they may not directly contribute to cardiovascular fitness or caloric burn, they are still beneficial for building muscle strength and stability. The ways in which the exercises improve QoL are stated by Liao et al. [16] as there are several mechanisms by which exercise can improve QoL and physical performance. First, engaging in exercise has the potential to enhance QoL by interacting with both biological and psychological systems. Specifically, exercise has been shown to increase cerebral blood flow, resulting in improved oxygen delivery to brain tissue and greater oxygen consumption. Other biological causes include decreased muscular tension, which improves cardiovascular health and can lead to increased endurance, strength, and overall physical fitness. Regular exercise can also improve muscle mass and bone density, which can decrease the risk of injury and improve overall physical performance. Second, exercise has been shown to have positive effects on mental health and emotional well-being in children and adolescents. Exercise can reduce stress and improve mood and self-esteem. These improvements in mental and physical health can lead to improved QoL scores. Finally, such improvement could also be attributed to proper adequate use of dialysis time with more attention from staff members. The effect of such exercises on the improvement of QoL is mostly multifactorial, which needs to be further investigated.

The current study denoted that there was no statistically significant correlation found between children’s age and their total QoL improvement after exercise. This means that no age group can benefit from exercise more than another, so all age groups could benefit from such an exercise program. However, specific programs should be tailored to each child individually according to their age, needs, medical condition, and fitness. Such programs could be increased gradually with time according to the tolerance of each patient. A physical therapist should be an integral part of each pediatric nephrology team and should be incorporated into the design of an individualized program for each patient.

Finally, the study revealed a significant association between QoL score and child level of education and the presence of other associated diseases (diabetes mellitus, hypertension, and heart failure) with kidney failure. These findings were in accordance with Darwish et al. [17], Abu-El-Goud et al. [18], and Baek et al. [19] who cited that the child’s self-reported total health score was significantly affected by their educational level and the presence of comorbid chronic diseases. It can be explained by the fact that a well-educated child is more aware of the quality of service and patient rights issues and, consequently, may be more demanding of the provided service. Comorbidities are common in children with CKD and can have a significant impact on their overall health and well-being. The presence of this associated disease can impact a child’s QoL in a number of ways, such as by increasing symptoms, limiting activities, and increasing the burden of treatment.

Actually, both dialysis-associated diseases, and kidney transplantation have a severe impact on the health-related QoL of children with kidney failure. Physicians should be aware of this continuous burden, and furthermore to develop tailored interventions for those children with kidney failure [20].

## Limitations

The study included a small sample size and the fact that it was conducted in a single-center setting, which may restrict generalizability. Also, the study included no objective testing of physical functioning other than the questionnaire answered by the patients on hemodialysis. It would be beneficial to incorporate objective testing of physical functioning alongside the self-reported measures. This could involve using validated assessments or physical performance tests to measure specific aspects of physical functioning relevant to the study population, such as muscle strength, endurance, or functional mobility. So, it would be advisable for future studies to incorporate objective measures alongside self-reported measures to strengthen the evidence and provide a more comprehensive evaluation of physical functioning in the study population. Another limitation of our study was that all the children included were on regular hemodialysis. Children on peritoneal dialysis were not included in the study, as peritoneal dialysis for kidney failure was not available in our country because of financial, environmental, and cultural causes. It is important for future studies to involve patients on peritoneal dialysis to consider the implications of the dialysis modality on the study results. Last but not least, reduction of the inactive time with more attention from the researchers to their patients during exercise may be responsible to a certain extent for the improvement of QoL. So future studies should consider this critical point to ensure that the increment in QoL testing is triggered by the “exercise” per se, not by better time manipulation or staff attention.

## Conclusion

The intensity of care for children on hemodialysis has a distinguished impact on their quality of life. The implementation of muscle stretching and isometric exercises during hemodialysis represents an important aspect of such care that may be associated with significant improvement in all domains of QoL. Children on hemodialysis need well-organized programs that cover all physical and psychological aspects with smart time manipulation and increased attention from their staff.

## Implications of the study

Healthcare organizations should encourage the use of physical exercise among children on hemodialysis, instruct healthcare providers on how to provide this, demonstrate proper techniques, and provide guidance on frequency and intensity. A health provider can also work with patients to develop individualized exercise plans that take into account their unique needs, abilities, and limitations. For future research, this study suggests the need for further well-designed randomized controlled trials of a multicentric nature with a larger sample size and exploring the use of technology to support patients in their exercise routines, such as through the use of mobile apps or wearable devices. Finally, the potential benefits of combining muscle stretching and isometric exercises with other interventions should be explored to enhance the overall QoL of patients.

## Recommendations

Muscle stretching and isometric exercises should become an essential component of routine clinical practice for children receiving hemodialysis to improve daily living activities and QoL. Also, health education should be given to parents about the importance of muscle stretching and isometric exercises, how to apply them, and encouraging them to do them with their children at home. Finally, developing a manual of guidelines for exercise programs and their benefits is recommended. This manual should be easily accessible within the unit and provided to newly admitted patients for a better demonstration of the importance of exercise. In addition, further studies on a larger population size, considering the duration of dialysis in years, different dialysis modalities, and objective assessment tools, are needed to confirm our results. Such future studies could explore the comparative effectiveness and patient satisfaction of home exercise programs versus exercise during dialysis sessions to inform best practices in this regard.

## Supplementary Information

Below is the link to the electronic supplementary material.

Graphical abstract (PPTX 550 KB)

Supplementary file2 (DOCX 74 KB)

## Data Availability

The datasets from this study are available from the corresponding author upon reasonable request.
