# Supplementary material for: Effect of muscle stretching and isometric exercises on quality of life in children undergoing regular hemodialysis
Source: Pediatr Nephrol. 2024 Jun 27;39(11):3289–99. doi: 10.1007/s00467-024-06398-2 (PMC11413026; doi:10.1007/s00467-024-06398-2)
Supplement: Supplementary file 1 — Graphical abstract (PPTX 550 KB) [file 467_2024_6398_MOESM1_ESM.pptx]

## Slide 1
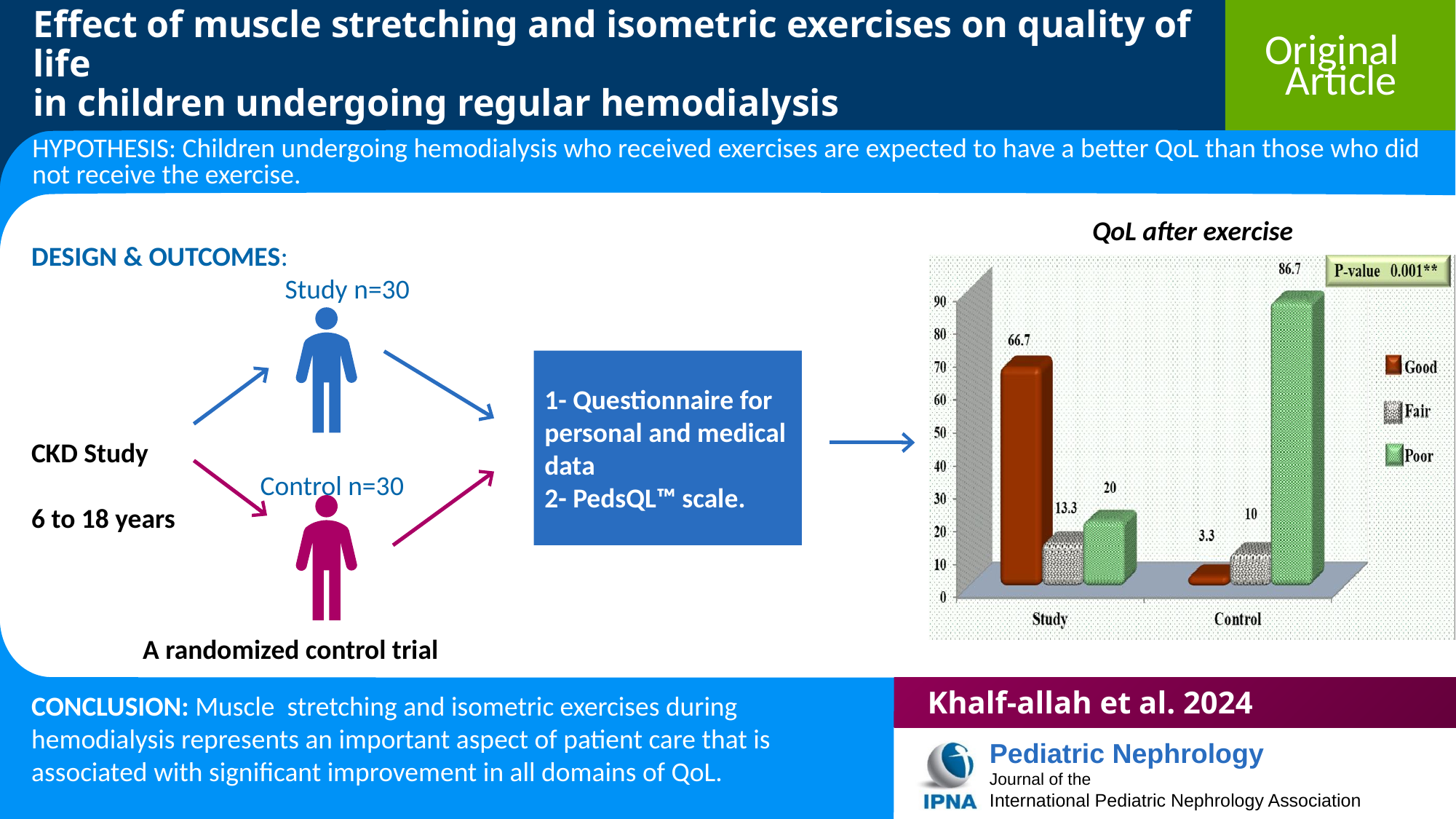

Effect of muscle stretching and isometric exercises on quality of life
in children undergoing regular hemodialysis
HYPOTHESIS: Children undergoing hemodialysis who received exercises are expected to have a better QoL than those who did not receive the exercise.
QoL after exercise
DESIGN & OUTCOMES:
 Study n=30
CKD Study
 Control n=30
6 to 18 years
 A randomized control trial
1- Questionnaire for personal and medical data
2- PedsQL™ scale.
Khalf-allah et al. 2024
CONCLUSION: Muscle stretching and isometric exercises during hemodialysis represents an important aspect of patient care that is associated with significant improvement in all domains of QoL.
