# Supplementary material for: Effect of muscle stretching and isometric exercises on quality of life in children undergoing regular hemodialysis
Source: Pediatr Nephrol. 2024 Jun 27;39(11):3289–99. doi: 10.1007/s00467-024-06398-2 (PMC11413026; doi:10.1007/s00467-024-06398-2)
Supplement: Supplementary file 2 — Supplementary file2 (DOCX 74 KB) [file 467_2024_6398_MOESM2_ESM.docx]

**Manuscript Title:** "Effect of Muscle Stretching and Isometric Exercises on Quality of Life in Children Undergoing Regular Hemodialysis"

Data of pilot study requested by reviewers :

**Table 1: Demographic data of the examined children n=6.**

| **Children’s personal data** | **Study group(n=3)** | | **Control group(n=3)** | | **P.value** |
| --- | --- | --- | --- | --- | --- |
|  | N | % | N | % |  |
| **Child age:** | | | | | |
| 6 <10 years | 2 | 66.7 | 2 | 66.7 | 0.8 |
| 10<14 years | 1 | 33.3 | 1 | 33.3 |  |
| 14:18 years | 0 | 0 | 0 | 0 |  |
| **Child gender:** | | | | | |
| Male | 2 | 66.7 | 2 | 66.7 | 0.8 |
| Female | 1 | 33.3 | 1 | 33.3 |  |
| **Child birth order:** | | | | | |
| 1^st^ | 0 | 0 | 2 | 66.7 | 0.18 |
| 2^nd^ | 1 | 33.3 | 0 | 0 |  |
| 3^rd^ | 0 | 0 | 0 | 0 |  |
| 4^th^ and more | 2 | 66.7 | 1 | 33.3 |  |
| **Child level of education:** | | | | | |
| Illiterate | 1 | 33.3 | 0 | 0 | 0.5 |
| Primary education | 2 | 66.7 | 3 | 100 |  |
| Preparatory education | 0 | 0 | 0 | 0 |  |
| Secondary education | 0 | 0 | 0 | 0 |  |
| **Consanguinity of parent:** | | | | | |
| Positive consanguinity | 2 | 66.7 | 1 | 33.3 | 0.5 |
| Negative consanguinity | 1 | 33.3 | 2 | 66.7 |  |

**Table 2: Allocation of examined children pertaining to their hemodialysis therapy n=6.**

| **Hemodialysis therapy** | **Study group(n=3)** | | **Control group(n=3)** | | **P.value** |
| --- | --- | --- | --- | --- | --- |
|  | N | % | N | % |  |
| **Cause of kideny failure:** | | | | | |
| Unknown cause | 0 | 0 | 1 | 33.3 | 0.5 |
| Congenital anomalies | 3 | 100 | 2 | 66.7 |  |
| Glomerulonephritis | 0 | 0 | 0 | 0 |  |
| Nephrotic syndrome | 0 | 0 | 0 | 0 |  |
| Lupus nephritis | 0 | 0 | 0 | 0 |  |
| **Associated diseases:** | | | | | |
| Yes(diabetes mellitus, hypertension and heart failure) | 2 | 66.7 | 1 | 33.3 | 0.5 |
| No | 1 | 33.3 | 2 | 66.7 |  |
| **Duration of hemodialysis therapy/ years**: | | | | | |
| <one year | 2 | 66.7 | 1 | 33.3 | 0.5 |
| 1<5 years | 1 | 33.3 | 2 | 66.7 |  |
| 5<10 years | 0 | 0 | 0 | 0 |  |
| 10 years | 0 | 0 | 0 | 0 |  |
| **Number of session per week:** | | | | | |
| Two sessions | 0 | 0 | 1 | 33.3 | 0.5 |
| Three sessions | 3 | 100 | 2 | 66.7 |  |
| **Duration of each session/ hour:** | | | | | |
| 3 hours | 0 | 0 | 1 | 33.3 | 0.5 |
| 4 hours | 3 | 100 | 2 | 66.7 |  |
| 5 hours |  |  |  |  |  |
|  | | | | | |
| **Complications during dialysis session:** | | | | | |
| Yes | 1 | 33.3 | 1 | 33.3 | 0.8 |
| No | 2 | 66.7 | 2 | 66.7 |  |
| **If yes** | | | | | |
| - Nausea and vomiting | 1 | 100 | 0 | 0 |  |
| - Dizziness, confusion and headaches | 0 | 0 | 0 | 0 |  |
| - Shortness of breath and chest pain | 0 | 0 | 0 | 0 |  |
| - Muscle cramps | 0 | 0 | 0 | 0 |  |
| - Hypotension | 0 | 0 | 0 | 0 |  |
| - Hypertension | 0 | 0 | 1 | 100 |  |

**Table 3: PedsQL™ scores before and after exercise n=6**

| **Quality of life aspects** | **Study group(n=3)** | **Control group(n=3)** | **P.value** |
| --- | --- | --- | --- |
|  | **Means ±SD** | **Means ±SD** |  |
| **Physical functioning** | | | |
| Before exercise | 208.33±200.52 | 325.0±294.74 | .601 |
| After exercise | 583.33±175.59 | 284.31±284.31 | 0.23 |
| **Emotional functioning** | | | |
| Before exercise | 191.66±123.32 | 291.66±236.29 | .021 |
| After exercise | 416.66±72.16 | 283.33±226.84 | .004 |
| **Social functioning** | | | |
| Before exercise | 200.00±108.97 | 316.66±236.29 | .016 |
| After exercise | 450.00±66.14 | 316.66±236.29 | .003 |
| **School functioning** | | | |
| Before exercise | 175.00±75.00 | 233.33±225.46 | .023 |
| After exercise | 408.33±57.73 | 233.33±225.46 | .007 |
| **Total score** | | | |
| Before exercise | 775.00±486.69 | 1166.66±988.15 | .022 |
| After exercise | 1858.33±321.45 | 1150.00±967.27 | .004 |

**Figure (1): Allocation of examined children pertaining to total QoL level before exercise n=6**

**Figure (2): Allocation of examined children pertaining to total QoL level after exercise n=6**

**Table 4: Relationship between children demographic and clinical data and QoL in the study group after exercise program n=6.**

|  | | **Child age** | **Child gender** | **Child birth order** | **Residence** | **Consanguinity of parent** | **Associated diseases** | **Cause of renal failure** | **Total Qol after exercise** |
| --- | --- | --- | --- | --- | --- | --- | --- | --- | --- |
| **Child age** | **r-value** |  |  |  |  |  |  |  |  |
|  | **p-value** |  |  |  |  |  |  |  |  |
| **Child gender** | **r-value** | **.250** |  |  |  |  |  |  |  |
|  | **p-value** | .633 |  |  |  |  |  |  |  |
| **Child birth order** | **r-value** | -.086- | -.086- |  |  |  |  |  |  |
|  | **p-value** | .872 | .872 |  |  |  |  |  |  |
| **Residence** | **r-value** | .000 | .000 | .485 |  |  |  |  |  |
|  | **p-value** | 1.000 | 1.000 | .329 |  |  |  |  |  |
| **Consanguinity of parent** | **r-value** | .000 | .000 | -.243- | -.333- |  |  |  |  |
|  | **p-value** | 1.000 | 1.000 | .643 | .519 |  |  |  |  |
| **Associated diseases** | **r-value** | .707 | .707 | -.485- | -.333- | .333 |  |  |  |
|  | **p-value** | .116 | .116 | .329 | .519 | .519 |  |  |  |
| **Cause of renal failure** | **r-value** | .316 | .316 | -.434- | -.447- | .447 | .447 |  |  |
|  | **p-value** | .541 | .541 | .390 | .374 | .374 | .374 |  |  |
| **Total Qol after exercise** | **r-value** | -.343- | -.343- | -.235- | .243 | .243 | **-.970-^**^** | -.759- |  |
|  | **p-value** | .506 | .506 | .654 | .643 | .643 | **.001** | .080 |  |
